# Impact of a Hypocaloric Diet on Prognostic Biomarkers of Endothelial Dysfunction: A Prospective Study

**DOI:** 10.3390/jcm15062321

**Published:** 2026-03-18

**Authors:** Cristina Lazar, Minela Aida Maranduca, Cristian Tudor Cozma, Andreea Clim, Mihaela Moscalu, Dragomir-Nicolae Serban, Ionela-Lacramioara Serban

**Affiliations:** 1 Faculty of Medicine, Grigore T. Popa University of Medicine and Pharmacy, 700115 Iasi, Romania; cristina-iuliana_lazar@umfiasi.ro (C.L.); tudor-cristian.cozma@umfiasi.ro (C.T.C.); clim.andreea@umfiasi.ro (A.C.); dragomir.serban@umfiasi.ro (D.-N.S.); ionela.serban@umfiasi.ro (I.-L.S.); 2 Internal Medicine Clinic, St Spiridon County Clinical Emergency Hospital, 700111 Iasi, Romania; 3 George I.M. Georgescu Institute of Cardiovascular Diseases, 700503 Iasi, Romania

**Keywords:** atherosclerosis, obesity, overweight, low-calorie, Castelli risk index, atherogenic index of plasma, lipid accumulation product

## Abstract

**Background/Objectives:** To assess the impact of weight loss on the atherogenic profile of patients with obesity, we proposed the Atherogenic Central Load Index (ACLI). The aim of the study was to validate ACLI as a novel lipid biomarker reflecting the balance between atherogenic and antiatherogenic lipoproteins, the overall atherogenic burden, and its association with inflammatory markers. **Methods**: A prospective study was conducted from January 2024 to July 2024. A total of 73 adults with overweight or obesity completed a six-month dietary-based weight loss intervention. A 15% caloric deficit target was set, excluding the potential influence of pharmacotherapy, and limiting physical activity to daily walking. Statistical analysis focused on anthropometric measures, lipid panel parameters and derived atherosclerosis indices. **Results**: The intervention returned a median weight loss of 11.8 (IQR: 8–19) kg. Before–after analysis showed a statistically significant improvement in anthropometric indices and most lipid profile components. To assess the effect of weight loss on the atherogenic profile of patients, we proposed an atherogenic load index (Atherogenic Central Load Index (ACLI)). ACLI decreased significantly following the hypocaloric diet and showed a significant correlation with the inflammatory markers hs-CRP and IL-6. ACLI showed a strong, inversely significant correlation (*p* < 0.05) with AIP, hs-CRP and IL-6, at the time of intervention initiation and after 6 months. The evaluation of the obtained AUC values allowed to clearly highlight the superior discrimination performance of ACLI regarding the inflammatory markers hs-CRP and IL-6 in patients with overweight and obesity involved in dietary interventions for weight loss. **Conclusions**. The proposed index (ACLI) showed strong and significant associations with key inflammatory markers, including hs-CRP and IL-6. Moreover, ACLI demonstrated superior discriminatory performance for elevated inflammatory status in overweight and individuals with obesity undergoing dietary weight-loss interventions, outperforming traditional atherogenic indices related to atherosclerosis progression (AIP, CRI–1, and CRI–2). These findings support the potential clinical utility of ACLI as an integrative marker of atherogenic burden and cardiometabolic risk.

## 1. Introduction

Despite the recent advancements in the diagnosis and management of cardiovascular disease (CVD) through the use of high-sensitivity biomarkers, microRNA detection and treatment targeting, as well as a decline in global age-standardized incidence, the global burden harbored by cardiovascular conditions remains the leading contributor to disability-adjusted life years (DALY) globally [1]. Regardless of sociodemographic conditions, high blood pressure, high low-density lipoprotein cholesterol (LDL-C) and ambient particulate matter are the primary risk factors attributable to DALY for CVD in individuals older than 55 years old [2]. Multiple satellite conditions, along with genetic and behavioral risk factors, interact synergistically to contribute to the advancement of cardiovascular disease. Often downplayed as a risk factor for the development of cerebrovascular disease or ischemic heart disease, atherosclerosis has been recently regarded as an early form of cardiovascular disorder by itself [3]. Atherosclerosis is a systemic progressive vascular disorder driven by a low-grade chronic inflammation (LGCI), leading to the accumulation of fatty fibrous material in the intimal layer of the small and medium arteries, mainly as a consequence of hyperlipidemia and lipid oxidation. This process is accelerated by an inflammatory response that involves the migration and proliferation of various immune cell types that are essential for the advancement of atherosclerotic plaques—neutrophils, T lymphocytes and macrophages [4]. Obesity is closely associated with the development and progression of atherosclerosis through a plethora of mechanisms, notably LGCI, insulin resistance, adipokine imbalance and adipocyte-derived exosome signaling [5,6].

Sedentary lifestyles and changes in the composition of food and beverages have given rise to a new epidemic in the 21st century: obesity. The development of obesity is multifactorial, and the contributing factors (behavioral, lifestyle, genetics, social, economic, and political factors) vary among individuals [7]. A major global health problem, especially in Western society, obesity is defined by the WHO as a body mass index (BMI) greater than or equal to 30 kg/m^2^, confirmed by at least one anthropometric criterion (waist circumference, waist-to-hip ratio, or waist-to-height ratio) in most scenarios [8]. Waist circumference, a reliable indicator of abdominal obesity (reference cutoff values for European populations: greater than 80 cm in women and 94 cm in men) and visceral fat accumulation are independent risk factors for cardiovascular disease which have been associated with higher mortality and morbidity rates [7,9].

The complex remodeling of adipose tissue found in patients with obesity translates into an altered secretion of adiponectin, leptin, and resistin, along with proinflammatory cytokines leading to LGCI, deeming adipose tissue an endocrine organ [10,11,12]. A tight balance of these hormones is involved in maintenance of cardiovascular homeostasis, inflammatory activity, insulin sensitivity, and adipose tissue balance. On the one hand, adiponectin protects against insulin resistance and metabolic syndrome; on the other hand, it enhances adipose tissue deposition, reduces thermogenesis, and is directly correlated with extent of inflammation [10]. Resistin levels serve as prognostic biomarkers for heart failure severity and mortality, while leptin levels are positively associated with an increased risk of heart failure [12,13]. In individuals with obesity, the disruption of adipokine regulation represents the foundation of endothelial dysfunction [14].

Obesity overrides the physiological anti-inflammatory milieu promoted by immune cells found in adipose tissue and those migrated in atheromatous plaques. An enhanced activation of “first responders”—neutrophils—and the increased migration of macrophages in the atheromatous plaques lead to upregulation of interleukin-6 (IL-6) and tumor necrosis factor-alpha (TNF-α). Along with excessive circulating levels of fatty acids, these trigger a self-sustaining permanent state of inflammation [15]. Compounding the high atherogenic and thrombotic risk, patients with obesity demonstrate increased thrombocyte count and platelet activity, as shown by the high concentrations of P-selectin and platelet-derived microparticles [16].

Given the intricate involvement of obesity on development and progression of endothelial dysfunction, we sought to understand the effect of mild dietary weight loss on several derived indexes previously associated with progression of atherosclerosis, the most prevalent form of early CVD in individuals with obesity.

The present findings support the clinical relevance of the Atherogenic Central Load Index (ACLI) as an integrative marker of cardiometabolic risk. Designed to increase with worsening atherogenic profiles—characterized by central adiposity, elevated triglycerides, and reduced HDL cholesterol—while limiting the influence of extreme triglyceride values, ACLI provides a stable estimate of overall atherogenic burden.

The significant pre–post changes observed following weight-loss intervention, together with its consistent correlations with established atherogenic indices (AIP, CRI–1, and CRI–2), support its validity as a comprehensive lipid-derived marker. Moreover, the observed associations between ACLI and key inflammatory markers (hs-CRP and IL-6) suggest that this index captures not only lipid-related risk but also the inflammatory component of atherosclerosis.

Taken together, these findings indicate that ACLI may represent a practical and clinically meaningful tool for monitoring the cardiometabolic benefits of weight reduction and for improving risk stratification in patients with overweight and obesity.

## 2. Materials and Methods

### 2.1. Study Design

This was a prospective study involving apparently healthy adults who voluntarily presented themselves to a private clinical practice during the beginning of 2024, aiming to lose body weight by dietary intervention alone.

This prospective observational study was conducted and reported in accordance with the STROBE (Strengthening the Reporting of Observational Studies in Epidemiology) guidelines. The completed STROBE checklist is provided in the Supplementary Material (Table S1).

Patients were subjected to a clinical examination by the main investigator, had a thorough check of their complete medical history, and had blood samples taken for a number of biochemical parameters listed below. Patient identification data was coded alpha-numerically. Blood results were interpreted independently by a second investigator, and the patients who fit the inclusion criteria were called for a second visit within 7 days to present them with the study protocol. Following informed consent being obtained, all subjects were assessed for intervention and invited for monthly physical follow-up visits. Blood samples were taken every two months (totaling four sets). At any moment, a patient could be withdrawn from the study if any of the following criteria applied: safety concerns in laboratory findings, new medical diagnosis overlapping with the exclusion criteria, deviations from protocol or a patient’s request to exit the study.

### 2.2. Timeframe

Patient recruitment extended over a period of 6 months, from 3 January 2024 to 1 July 2024. The patient follow-up period was fixed at 6 months, regardless of enrollment date. The study officially ended on 30 December 2024.

### 2.3. Inclusion and Exclusion Criteria for Enrollment

We included adults aged between 18 and 65 years old with an excess ponderal status (established by BMI and at least one other anthropometric measurement) who presented voluntarily to a private clinical practice. The patients included in the study had BMI values ranging from 30.4 to 42.1 kg/m^2^.

Exclusion criteria: stable residence outside a 50 km radius from the city, lack of motivation, current medical therapy interfering with weight loss, serious physical impairments (walking with aid, requirement of permanent assistance), significant medical history (history of cerebrovascular ischemic disease; coronary artery disease; heart failure stage III–IV NYHA; pulmonary hypertension; any mild, moderate or severe valvular disease; history of peripheral artery disease; trauma leading to severe motor deficits, any severe musculoskeletal condition which could interfere with walking ability; documented chronic kidney disease stage IIIa or higher; history of chronic liver disease; moderate or severe pulmonary disease or insufficiently controlled mild pulmonary disease; diabetes of any type; Instrumental Activities of Daily Living score less than 8; recent or ongoing psychiatric treatment, recently or currently suffering from any oncological disease; high clinical suspicion of sarcopenia (gait speed less than 0.9 m/second, handgrip strength less than 25 kg in men, and 18 kg in women); abnormalities in the physical examination (signs of active infectious disease, fever during last 48 h, pain of one major joint, or at least three minor joints, chest pain regardless of characteristics, breathing difficulties after 5 attempts in the chair-sit-to-stand test); and abnormalities in the blood screening panel (fasting plasma glycemia over 100 mg/dL, glycated hemoglobin over 5.7%, estimated glomerular filtration rate less than 60 mL/min/1.73 m^2^, white blood cells over 10.000/µL, hemoglobinemia lower than 12 g/dL in women and 14 g/dL in men, platelet count lower than 150.000/mm^3^).

### 2.4. Control of Bias and Confounding

Potential sources of error were minimized throughout the study. Selection bias was reduced by applying predefined inclusion and exclusion criteria and by consecutively recruiting eligible patients throughout the study period. All participants were enrolled from the same clinical setting and were followed up according to a standardized protocol.

Information bias was minimized through the use of standardized measurement procedures and uniform data collection. Anthropometric parameters and blood pressure were assessed by trained medical personnel using calibrated equipment, while biochemical analyses were performed in the same certified laboratory using validated methods. Data were recorded using a predefined case report format to ensure consistency.

Potential confounding was addressed by collecting relevant demographic, clinical, and metabolic variables, including age, sex, baseline anthropometric measures, and metabolic parameters. In addition, the pre–post study design allowed each participant to serve as their own control, thereby reducing the influence of inter-individual variability.

**Figure 1:**
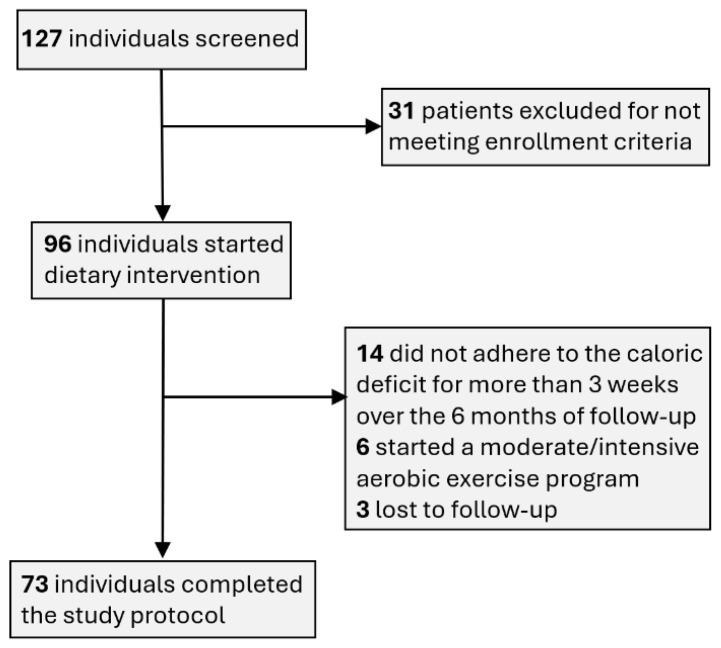
Selection of study participants.

A modest proportion of the subjects enrolled were withdrawn over the course of the study, with the reasons being patient safety, acquirement of a serious medical condition, deviation from intended intervention and patient’s request. As shown in Figure 1, 6 patients actively pursued moderate-to-intensive aerobic physical activity regimens, which contradicts with our dietary weight loss intervention; 14 patients did not stick to the required 15% intake deficit during more than 3 weeks over the course of the study; and 3 patients were lost to follow-up. There were no cases of safety concerns being flagged during periodic physical examinations or by the blood panel interpretation.

### 2.5. Intervention

The study did not involve any pharmacological interventions and did not interfere with any of the patients’ ongoing prescribed medications. The recommended physical activity was 8000–10,000 steps daily, at a slow–moderate pace, without any intense aerobic efforts. Daily step counts were monitored using personal smartphones or wearable activity trackers, and participants reported their average daily step counts during follow-up visits.

Maintenance calories were estimated at enrollment, using the Mifflin St Jeor equation (men: basal metabolic rate = (10 × weight in kg) + (6.25 × height in cm) − (5 × age in years) + 5; women: basal metabolic rate = (10 × weight in kg) + (6.25 × height in cm) − (5 × age in years) − 161) and standard activity factor taking values of either 1.2 (in sedentary individuals) or 1.375 (in those reporting some exercise weekly). To achieve a caloric intake reduction of 15%/day, the participants were advised to record either the ingredient composition of the in-house prepared food by using a kitchen scale, or track the calories listed in every already-cooked meal. General dietary advice was given in printed form to all participants, advising manageable methods to decrease overall calorie intake (e.g., restricting the consumption of processed, fat-rich foods, calorie-dense low-filling meals), to increase their protein intake to 1 g/per kg body weight, as well as their dietary fiber consumption, and to consume at least 2000 mL of water daily. Compliance with the dietary intervention was reviewed every 7–10 days using patients’ daily food diaries and weekly weight journals. Concerning physical activity, all patients were advised to achieve between 8000 and 10,000 steps per day, with their weekly records being transmitted to the principal investigator at the same moment as the calorie intake journal. This was considered a negligible component for weight loss in the context of the ensuing 15% calorie deficit.

### 2.6. Anthropometric and Biochemical Parameters Recorded

After pseudonymization of the patient identification data, we recorded a set of anthropometric measurements: age, biological gender, weight, waist circumference, and daily activity level. The biochemical parameters recorded at enrollment and study completion: hemoleucogram (along with derived erythrocyte parameters, leucocyte formula), lipid profile (total cholesterol (TC), high-density lipoprotein cholesterol (HDL-C), low-density lipoprotein (LDL-C), triglycerides), glycemic profile (fasting plasma glucose, glycated hemoglobin (HbA1c)), liver function panel (aspartate aminotransferase AST, alanine aminotransferase ALT, gamma glutamyl transferase GGT, bilirubin levels), and renal function (urea, creatinine).

The inflammatory markers high-sensitive C-reactive protein (hs-CRP), Interleukin-6 (IL-6) and lactate dehydrogenase LDH were also recorded. The two blood panels at 2 and 4 months after enrollment comprised hemoleucogram, glycemic profile, and liver and renal function tests. Of all these variables, only the most relevant ones are presented in this study to highlight the research conclusions.

### 2.7. Planned Outcomes

Derived indices associated with atherosclerosis progression were calculated: atherogenic index of plasma (AIP), Castelli Risk Indexes–1 (CRI–1) and Castelli Risk Indexes–2 (CRI–2), and lipid accumulation product (LAP), using the standard formulas:

(1) $$AIP={log}_{10}\left(\frac{Triglicerides}{HDL\;cholesterol}\right)$$

(2) $$CRI–1=\frac{Total\;cholesterol\;}{HDL\;cholesterol}$$

(3) $$CRI–2=\frac{LDL\;cholesterol\;}{HDL\;cholesterol}$$

(4) $${LAP}_{male}=\left(waist\;circumference\;[cm]-65\right)\times triglicerides\;[mmol/L]\;$$

(5) $${LAP}_{female}=\left(waist\;circumference\;[cm]-58\right)\times triglicerides\;[mmol/L]$$

To assess the effect of weight loss on the atherogenic profile of patients, we proposed an atherogenic load index (Atherogenic Central Load Index (ACLI)). The proposed index considers several important benchmarks: its value should increase when the profile becomes highly atherogenic (large waist, high triglycerides, low HDL) and should not be influenced by extreme triglyceride values.

The proposed formula for ACLI is:

(6) $${ACLI}_{male}=\left({WC}_{\left[cm\right]}-65\right)\times ln\left(1+\frac{{Triglicerides}_{mmol/L}}{{HDL\;cholesterol}_{mmol/L}}\right)$$

(7) $${ACLI}_{female}=\left({WC}_{\left[cm\right]}-58\right)\times ln\left(1+\frac{{Triglicerides}_{mmol/L}}{{HDL\;cholesterol}_{mmol/L}}\right)$$

This ensures coherence from a pathophysiological point of view, and logarithmic transformation of the ratio was applied to ensure robustness of the formula to extreme values. In other words, the formula has a reduced sensitivity to outlier values.

At the same time, the proposed formula applies a normalization of the index by referring to the protective component of HDL values that is found in the AIP formula. To quantify the change induced by the intervention in absolute values, we calculated Δ ACLI = ACLI_POST_ − ACLI_PRE_. To validate the new index, we evaluated the pre–post intervention change and performed its correlation analysis with the traditional indices: AIP, CRI–1 and CRI–2. We also tested the correlation of the new index with the values of the inflammatory markers hs-CRP and IL–6.

### 2.8. Statistical Analysis

The statistical analysis of data was performed using SPSS v.29.0 (IBM Ireland Product Distribution Limited, IBM House, Shelbourne Road, Ballsbridge, Dublin 4, Ireland) and the STATA 16 software (StataCorp LLC, 4905 Lakeway Drive, College Station, TX 77845-4512, USA). The qualitative variables were presented as absolute (n) and relative (%) frequencies. Descriptive statistics include absolute numbers, percentages, mean, standard deviation (SD), median, and interquartile range (IQR), in accordance with the variable type and distribution symmetry. The Kolmogorov–Smirnov test was used to verify whether the distribution of continuous variables was normal. The t-test for dependent samples or Wilcoxon matched-pairs test were applied to compare continuous variables, depending on the type of distribution of each. To highlight the predictive value of the atherogenic profile based on the new proposed index (ACLI) compared to the traditional indices, Pearson correlation tests were performed and the correlation coefficients (r), and the slopes of the regression lines were compared according to the regression line equation.

The accuracy of the predictive power was comparatively evaluated based on the receiver operating characteristic (ROC) curve, taking into account the area under the curve (AUC), which represented the compromise between the sensitivity (Se) and specificity (Sp) of the method used. The significance level calculated in utilized tests (*p*-value) was considered significant for the values of *p* < 0.05.

### 2.9. Ethical Approval

Upon study enrollment, all patients gave their informed consent, allowing limited use of their anthropometric and laboratory data, in agreement with Declaration of Helsinki and local ethical regulations. Ethical approval was received from the Scientific Research Committee of Grigore T. Popa University of Medicine and Pharmacy in Iasi, Romania (345/7 September 2023). The Public Health Directorate of Iasi, in accordance with national regulations, waived the need for ethical approval due to the design of the study.

### 2.10. Sample Size Calculation

To determine the optimal sample size, we calculated the minimum volume required to ensure the representativeness of the patient category and used relevant data from the literature [17,18]. To achieve this prerequisite, we established a 95% confidence interval.

Accordingly, we used the equation: $n\geq {\left(Z_{\left(1\;-\;\frac{\alpha }{2}\right)}\right)}^{2}\times \frac{\sigma^{2}}{d^{2}}$ with $Z_{\left(1\;-\;\frac{\alpha }{2}\right)}=1.96$ for a 95% confidence interval and an allowed error of 2% (d = 0.02). For a standard deviation (σ) of 8.59% for weight loss, the minimum sample size should be 71 cases (n_calculating_ ≥ 70.87).

Thus, in this research, from the total number of patients (127 patients) and applying the inclusion and exclusion criteria, a study group with 73 cases resulted.

## 3. Results

**Figure 2:**
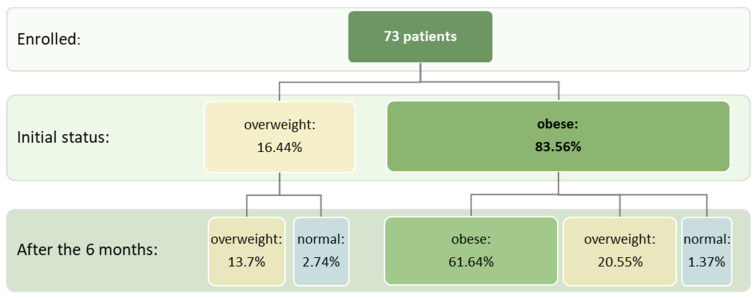
Evolution of ponderal status before and after intervention.

Out of the 73 patients who managed to complete the intervention protocol, in the enrollment phase, 12 (16.4%) were overweight and 61 (83.6%) were obese. Looking at the number of cases in each patient group (overweight, obese), it was revealed that after 6 months, 10 out of the 12 overweight patients (83.3%) and 45 out of the 61 patients with obesity (73.8%) remained in the same anthropometric category. Only two initially overweight patients (16.7%) and one patient with obesity (1.7%) advanced to the normal category (BMI and WC). A total of 15 out of the initial 61 individuals with obesity advanced to the overweight category (24.6%). These changes are presented in Figure 2, which shows the relative frequencies reported for the total number of patients included in the study (73 patients).

The 73 individuals lost a median of 11.8 kg over the study period (IQR: 8–19 kg). Notably, 25 subjects (34.2%) lost less than 10% of their starting body weight, with the cutoff value agreed for efficiency of the intervention. A summary of anthropometric and biochemical characteristics of interest at the two points is depicted in Table 1. Evaluation of the results indicated that the only parameter that did not change significantly post-intervention was CRI–1 (*p* = 0.054).

ROC for AIP as prognostic factor for obesity returned a statistically significant value (AUC = 0.710, 95%CI: 0.567 to 0.854), with a cutoff value for best accuracy at 0.30. This relationship did not extend beyond the enrollment time point. LAP was an accurate prognostic factor for distinguishing patients with obesity from individuals with overweight both at baseline and study exit (AUC = 0.883, 95%CI: 0.800 to 0.965, and exit (AUC = 0.857, 95%CI: 0.771 to 0.943).

The dietary weight loss intervention led to a statistically significant difference in three out of the five main outcomes recorded: AIP, CRI–1, CRI–2, LAP and ACLI (Table 2). While AIP showed improvement (i.e., lower values) following intervention, the difference did not attain statistical significance, the results indicating a statistical uncertainty (*p* = 0.046). CRI–1 showed virtually no difference between the two time slots (Table 1).

In this context, the research aims to validate an index, called Atherogenic Central Load Index (ACLI), as a new lipid biomarker that comprehensively evaluates the balance between atherogenic and antiatherogenic particles in the blood to effectively reflect the cumulative atherogenic effect and its association with inflammatory markers. To highlight the value of the new index, we evaluated the pre–post intervention change and performed correlation analysis with the traditional AIP, CRI–1 and CRI–2 indices. We also tested the correlation of the new index with the values of the inflammatory markers hs-CRP and IL–6.

**Figure 3:**
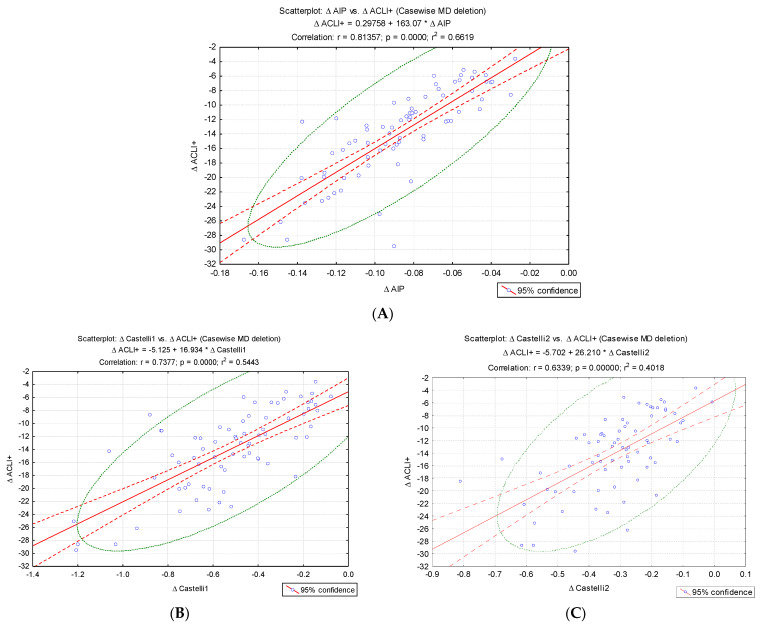
Correlations of the variations (Δ) atherogenic indices: (**A**) Δ AIP, (**B**) Δ CRI–1, (**C**) Δ CRI2 and Δ ACLI values.

To validate the new index, we evaluated the pre–post intervention change in ACLI values (Δ ACLI) and performed a correlation analysis of the differences recorded in the evolution with the changes in Δ AIP, Δ CRI–1 and Δ CRI–2 (Figure 3). A significant increase in Δ ACLI was noted with the changes in the values of the traditional atherogenic indices: Δ AIP (r = 0.81, *p* < 0.001), Δ CRI–1 (r = 0.73, *p* < 0.001) and Δ CRI–2 (r = 0.63, *p* < 0.001) (Figure 3).

**Figure 4:**
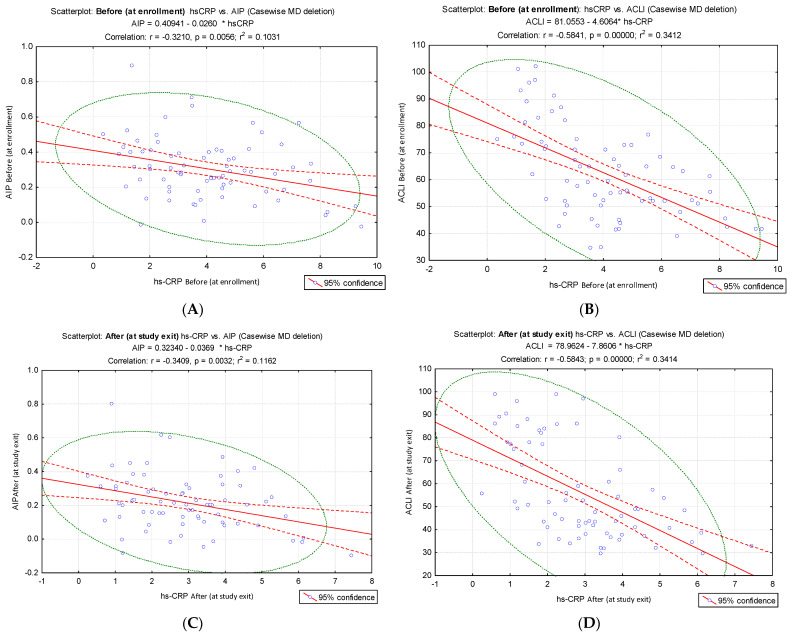
Correlation of hs-CRP values from pre- and post-intervention times with AIP and ACLI values, respectively. (**A**) Pre-intervention (before) hs-CRP vs. AIP; (**B**) pre-intervention (before) hs-CRP vs. ACLI; (**C**) post-intervention (after) hs-CRP vs. AIP; (**D**) post-intervention (after) hs-CRP vs. ACLI.

Given that endothelial dysfunction is characterized by pro-inflammatory status, decreased vasodilation, and increased vasoconstriction, the study demonstrated a significant correlation of the proposed index (ACLI) with the inflammatory markers hs-CRP and IL-6. To allow a comparison of the intensity of the link between ACLI and AIP and the inflammatory markers hs-CRP and IL-6, we analyzed the results of the correlation tests. We compared the correlation coefficients and the slopes of the corresponding regression lines. The results indicated a stronger inverse correlation of hs-CRP values with ACLI values (before: hs-CRP vs. ACLI, r = −0.58, *p* < 0.001, Figure 4B; after: hs-CRP vs. ACLI, r = −0.58, *p* < 0.001, Figure 4D) compared to the results of the correlation of hs-CRP vs. AIP (before: hs-CRP vs. AIP, r = −0.32. *p* = 0.0056, Figure 4A; after: hs-CRP vs. AIP, r = −0.34, *p* = 0.0032, Figure 4C).

**Figure 5:**
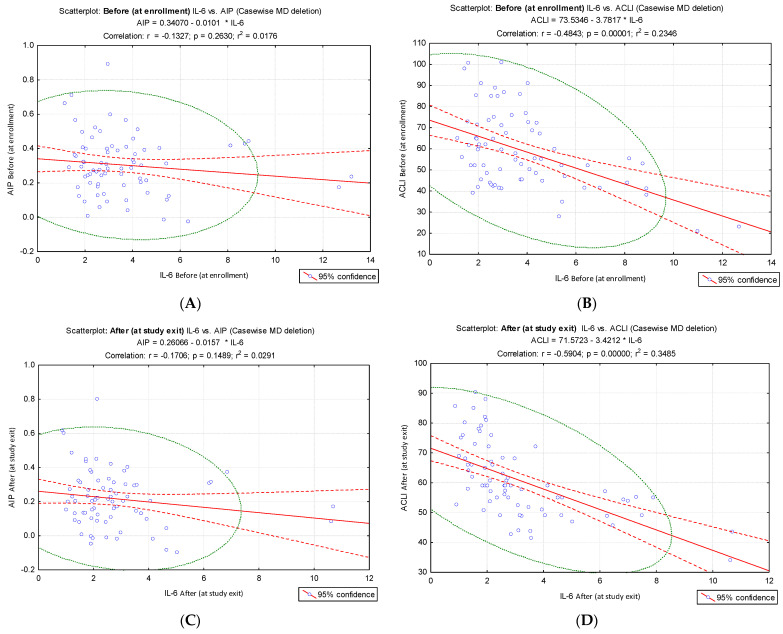
Correlation of IL-6 values from pre- and post-intervention times with AIP and ACLI values, respectively. (**A**) Pre-intervention (before) IL-6 vs. AIP; (**B**) pre-intervention (before) IL-6 vs. ACLI; (**C**) post-intervention (after) IL-6 vs. AIP; (**D**) post-intervention (after) IL-6 vs. ACLI.

Different results were obtained when IL-6 values were correlated with AIP and ACLI values at the two evaluation times, before: IL-6 vs. ACLI, r = −0.48, *p* < 0.001, Figure 5B; after: IL-6 vs. ACLI, r = −0.59, *p* < 0.001, Figure 5D), compared to the results of the IL-6 vs. AIP correlation (before: IL-6 vs. AIP, r = −0.13. *p* = 0.263, Figure 5A; after: IL-6 vs. AIP, r = −0.17, *p* = 0.149, Figure 5C). IL-6 values at the time of enrollment and at the end of the intervention did not show a significant correlation with AIP, but they showed a significant inverse correlation with ACLI.

**Figure 6:**
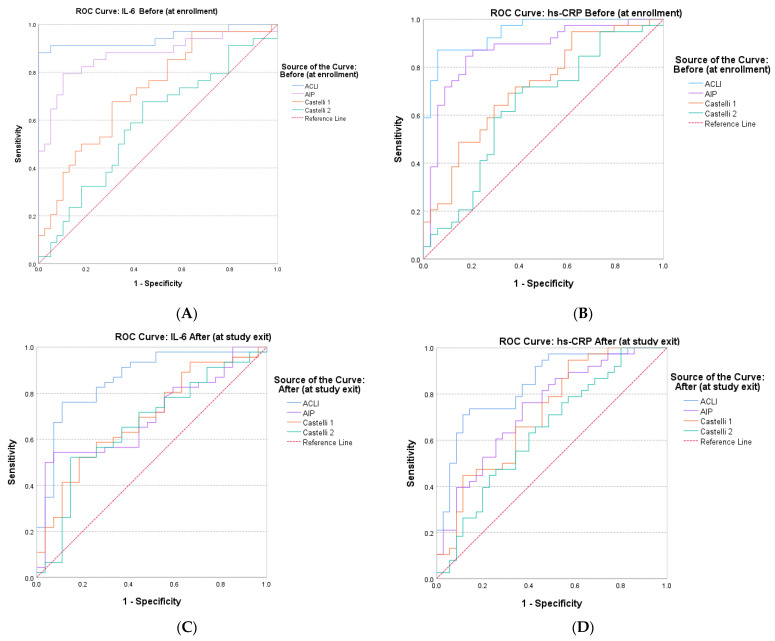
Receiver operating characteristic (ROC) curves for comparative evaluation of the predictive value of AIP, CRI–1, CRI–2 and ACLI for the inflammatory markers hs-CRP and IL-6. (**A**) Preintervention (before) IL-6; (**B**) pre-intervention (before) hs-CRP; (**C**) post-intervention (after) IL-6; (**D**) post-intervention (after) hs−CRP.

The results obtained provide a clear conclusion on the increased predictive value that the new index (ACLI) has for the inflammatory markers hs-CRP and IL-6. To comparatively evaluate the accuracy that the atherogenic indices (AIP, CRI–1, CRI–2 and ACLI) have in predicting inflammatory markers, we analyzed the ROC curves (Figure 6) and AUC values (Table 3).

Analysis of the obtained AUC values demonstrated the superior discriminatory performance of ACLI for the inflammatory markers hs-CRP and IL-6 in patients with overweight or obesity undergoing dietary weight-loss interventions. The AUC values for ACLI were significantly higher than those corresponding to the other atherogenic indices evaluated (AIP, CRI–1 and CRI–2) for both evaluation times (before intervention and after intervention).

All our results highlighted the superior potential of ACLI for predicting endothelial dysfunction with reference to the inflammatory markers hs-CRP and IL-6, compared to traditional derived indices associated with atherosclerosis progression (AIP, CRI–1 and CRI–2).

**Figure 7:**
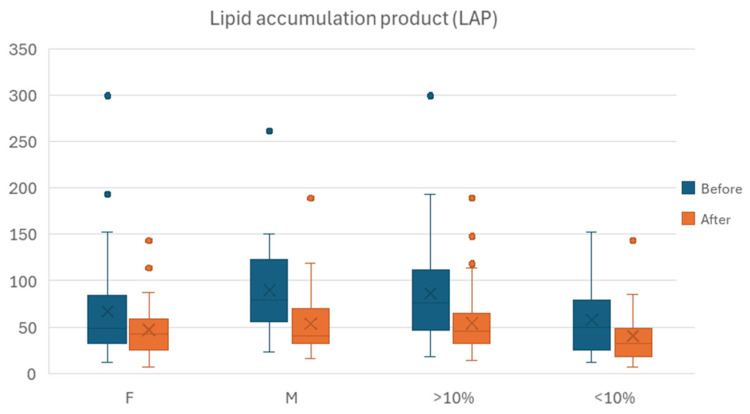
Evolution of LAP before–after comparison wise, stratified by percentage of body weight lost (BWL), with lower than 10% classified as mild, more than 10% as moderate; and by gender (M, men; F, women).

LAP kept the statistically significant difference observed for the entire population for before–after assessment in both females and males, with the lower values recorded at the latter point in time. Figure 7 illustrates the differences in outcomes.

**Figure 8:**
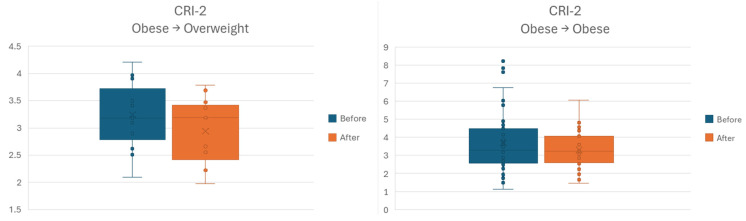
Before–after comparison stratified by classifying individuals by body weight category at study entry and exit, respectively. CRI–2, Castelli Risk Index–2.

For CRI–2 outcome, the before–after comparison showed lower values at the latter time point for both categories of individuals starting as obese. However, sample size only allowed for the larger subgroup to attain a statistically significant difference, as shown in Figure 8.

The assessment of insulin resistance and the analysis of its relationship with the proposed (ACLI) provide additional insights and support its metabolic validation. This study is currently ongoing and represents the final stage in the validation of ACLI as a potential indicator of insulin resistance. The atherogenic index proposed in this study may reflect insulin resistance, a central metabolic disorder closely involved in the process of atherogenesis. Insulin resistance is characterized by a typical dyslipidemic profile, including elevated triglyceride levels, reduced HDL cholesterol, and a predominance of small, dense LDL particles, all of which contribute to increased atherogenic potential. Beyond lipid abnormalities, insulin resistance is associated with endothelial dysfunction, chronic low-grade inflammation, oxidative stress, and vascular smooth muscle cell proliferation. These mechanisms promote the initiation and progression of atherosclerotic plaques. Therefore, the observed relationship between the proposed atherogenic index and cardiovascular risk supports its potential role as an indirect marker of insulin resistance and its proatherogenic effects.

## 4. Discussion

**Figure 9:**
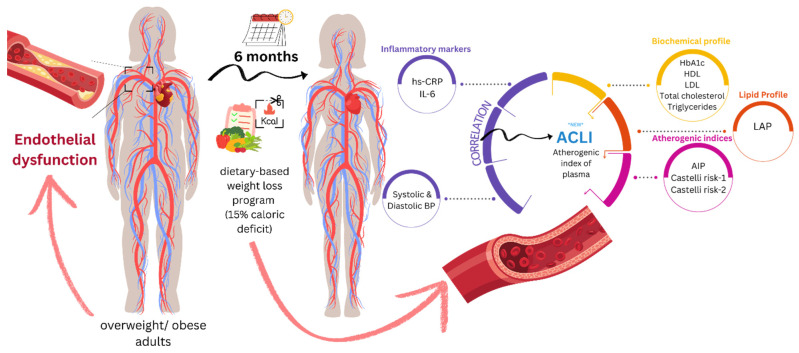
Mechanisms underlying endothelial dysfunction in obesity or overweight.

For an index to be relevant to our study, it should be associated with the progression of atherosclerosis, an early manifestation of CVD commonly observed in patients with overweight or obesity. The literature reports numerous indicators for predicting CVD progression or mortality, ranging from indices derived from combinations of laboratory and/or anthropometric parameters, to markers based solely on laboratory findings, and ratio-based indicators. Product-type-derived indicators, where all component parameters are expected to shift in the same direction, did not require additional proof of evidence through our research. Therefore, we included the AIP, CRI–1 and CRI–2 ratio-type indexes. LAP was added to the outcomes, despite the product-type formula, to find which confounding factors might influence the change in LAP throughout the diet-based weight loss intervention. In line with the ratio-type indexes, and inspired by LAP, we included a Atherogenic Central Load Index (ACLI), where we kept the same waist correction. A graphical summary of the research objectives is presented in Figure 9.

### 4.1. Atherogenic Index of Plasma

The atherogenic index of plasma, with the classical formula AIP = log10(TGL/HDL-C), proved a statistically significant predictor ability for excess weight status at enrollment when comparing patients with overweight and those with obesity. While no clear normal range has been defined, AIP values under 0.11 are considered to yield lower risk for cardiovascular disease [19]. In our study, less than 5% of patients both in the initial and final settings achieved this lower threshold. AIP did not prove a relevant association to absolute values for BMI or body weight, but was directly correlated to triglyceride levels, and inversely correlated to HDL-C at enrollment. These findings are in line with the existing literature in both general and selected populations, with studies reporting a better prognostic capacity of AIP for excess body weight in comparison to other simple laboratory lipid values [20,21]. In larger adult populational samples, AIP has proven a statistically significant direct correlation with BMI and complementary anthropometric measurements such as waist circumference and waist-to-hip ratio [22,23]. Stratifying for AIP values, HDL-C and TGL showed opposing trends, with the highest quartile values of AIP returning lower HDL-C and higher TGL values and vice versa [21,22].

In our study, the intervention of dietary weight loss did not return a statistically significant difference for AIP values, neither for the entire population sample, nor for subgroups generated by percentage of weight lost, by gender stratification, or by change in category. These findings align with the existing literature, in the manner that an efficient weight drop does not necessarily translate into an improvement in AIP [24]. A more performant variant of AIP restricted to only the most anti-atherogenic fractions of HDL-C has been proposed to the scientific community, but the interest in it is still behind most modified indices (AIP-WC, AIP-waist-to-height-ratio, AIP-BMI). Other studies, however, indicate that a reduction in AIP parallels the improvement in body weight, as shown in diabetic patients after bariatric surgery or after treatment with a bergamot polyphenol extract complex, with the method employed for weight loss being of lesser importance [25,26].

The importance of this index stems from its widespread associations in general population cohorts with progression of cardiovascular disease (incidence of heart failure, progression of atherosclerotic coronary disease) and incidence of major adverse cardiovascular events and cardiovascular death [27,28,29]. Not only the individuals with alleged coronary artery disease (as evidenced by angiography) demonstrate association between elevated AIP and increased incidence of cardiovascular events, but also patients suffering from microvascular disease, such as type 2 diabetes mellitus or cardio-vascular-kidney metabolic syndrome [30,31]. The evidence suggests that lower values of AIP in the general population correlate with a reduced risk for major adverse cardiovascular events, after adjustment for traditional CVD risk factors [32]. Our selected study population is a close approximation for the typical individual fit for a weight loss intervention.

AIP appears to be a better prognostic factor for insidious atherosclerotic cardiovascular disease in the general population, compared to other lipid-based composite indices [33]. In addition, evaluation of AIP at hospital admission for acute myocardial infarction is an accurate predictor for all-cause mortality over one-year follow-up [34]. Presumably, the connecting link between high AIP values and increased risk of CVD is represented by traditional cardiovascular risk factors, such as obesity, elevated blood pressure, elevated fasting plasma glucose, and oxidative stress, but proof remains heterogeneous [22,23,35,36].

### 4.2. Castelli Risk Index–1 and–2

We used the common formula CRI–1 = TC/HDL-C for Castelli Risk Index–1 and CRI–2 = LDL-C/HDL-C for Castelli Risk Index–2. Within the whole sample, the difference between the patients with overweight and those with obesity was not statistically significant at either enrollment or study completion for both indices. Comparison of the before and after intervention values returns lower values for both indices after the weight loss procedure, which hold statistical significance only for the CRI–2 parameter. Subgrouping for body weight loss, gender or anthropometric category change resulted in a clinically and statistically non-significant difference in any of the resulting subgroups for CRI–1. A similar subgroup analysis for CRI–2 in the baseline–after comparison revealed a statistically significant difference in the male and obesity subgroups. Considering the accepted range of normal values for both indexes, integrating both CRI–1 and CRI–2 values into a single dichotomous index (defined as modified if any of the two is elevated), our intervention resulted in a 10.5% reduction in the number of patients at increased risk for cardiovascular disease—from 52.1% above the upper limit at inclusion, to 46.6% at exit [19]. In our study, there was a strong correlation between the relative changes in CRI–1 and CRI–2. This is probably due to the significant fraction of total cholesterol present in the LDL form, whereby any variation in total cholesterol corresponds directly to a change in LDL cholesterol.

The presence of metabolic syndrome and increased body weight in comparison to normal weight have been associated with increased values in both indices [37,38]. However, our study lacked a control group, and no statistically significant differences were observed between individuals with overweight and those with obesity either at enrollment or at the end of the study. Weight loss after bariatric surgery in patients suffering from type 2 diabetes led to a significant improvement in HDL-C and a statistically significant reduction in AIP, CRI–1 and CRI–2 over the long-term [25]. Another study concerning the effects of bariatric surgery upon improvement of metabolic profile showed that CRI indices were positively correlated to weight loss and inversely to excess fat mass [39]. To our knowledge, this is the first study concerning the effect of dietary-based weight loss intervention upon indices for atherosclerosis and advanced cardiovascular disease. The important reduction in CRI–2 translates to improved cardiovascular health and delayed CVD installation.

Atherosclerotic cardiovascular disease, an early manifestation of CVD, can be analytically predicted through the use of CRI–1 and CRI–2. Both exhibited sensitivities exceeding 70% in a study involving 298 adults at a tertiary care center. Adjustment for several anthropometric and clinical factors (e.g., age, smoking status, presence of diabetes mellitus, frequency of physical activity practice) canceled the predictive ability of CRI–1 and CRI–2, contrasting with the robustness of AIP [33]. CRI–1 and CRI–2 held prognostic significance for any form of CVD, but in that case the association appeared more robust in women from the general population [40]. Another piece of research noted that CRI–1 and CRI–2 may display sex-related differences, since both returned higher values in men of working age before and after adjustment for anthropometric and laboratory parameters [41]. This observation may constitute the basis for the noted epidemiological differences in cardiometabolic disease, warranting the use of these indices in fundamental screening.

Presence and severity of coronary artery disease (CAD) as predicted by CRI–1 and CRI–2 is still disputed. Angiographically confirmed presence of CAD was associated with higher values of these indices, in spite of the absence of a difference in lipid profile lab values [42]. This finding was confirmed in a more recent study on 1187 subjects, 781 of which underwent coronary angiography. Not only was presence of stenosis associated with presence of arterial stenosis, but an individual increase in CRI–1 or CRI–2 was associated with multi-vessel coronary artery stenosis [43]. However, the predictive ability may be voided under acute settings. Evaluation of cardiometabolic composite indices within the near-time proximity to an acute myocardial infarction—with or without ST elevation—event does not show promising prognostic features [34,44]. In patients who suffered such a major cardiovascular event (i.e., MACE, a form of manifest CAD), neither CRI–1 nor CRI–2 should be used as predictors for another MACE or all-cause mortality over long-term follow-up [34].

### 4.3. Lipid Accumulation Product and Atherogenic Central Load Index

In our study we calculated LAP using a formula for women (waist circumference [cm] − 58) × (triglycerides [mmol/L]) and for men (waist circumference [cm] − 65) × (triglycerides [mmol/L]), advanced over 20 years ago for a better prognostic ability compared to BMI for cardiovascular risk [45]. Diet-based approach to weight loss in our study resulted in a reduction in LAP, statistically significant after adjustment for several factors—gender, percentage of weight lost, anthropometric starting and ending category. The effect of controlled appropriate diet upon improvement of lipid profile has been demonstrated in both healthy and metabolically impaired adults. Evidence shows that adopting a DASH diet (Dietary Approaches to Stop Hypertension) in adults with metabolic syndrome can significantly improve lipid profile [46]. A more permissive strategy based on semiquantitative assessment of the subjects’ usual diet showed that a lower carbohydrate content is associated with reduced levels of visceral fat, although this relation was true only in women [47].

Within the before–after analysis, we performed stratification for percentage of body weight loss as a result of the intervention, using the cutoff value of 10% This resulted in a 25-individual moderate weight loss sample and a 48-individual mild weight loss group. AIP and CRI–1 showed no statistically significant difference within any of the two resulting subgroups between the two instances of time. While CRI–2 returned a statistically significant difference between study exit and enrollment for the whole sample, stratification by relative weight loss canceled the statistical significance in both resulting subgroups.

LAP maintained the statistically significant difference observed for the entire population for before–after assessment in both of the resulting subgroups, with the lower values recorded at the latter point in time.

We also performed stratification for gender, male and female, as medically assessed at enrollment. Despite AIP did not return a statistically significant difference between the before and after time points for the overall sample. Only the male group showed lower overall values for AIP outcome, the difference attaining statistical significance. Neither the male nor the female subgroup showed a significant before–after difference for the CRI–1 outcome. For the CRI–2 outcome, however, stratification for gender maintained statistical significance for the before–after difference within the male subgroup.

The last stratification attempt was performed for change (or lack thereof) of anthropometric category. The sample size allowed the identification of three subgroups: 10 individuals with overweight who remained overweight, 15 individuals with obesity who moved to the overweight category, and 45 individuals who remained obese. In neither of the three groups for outcomes AIP and CRI–1 was the difference between before–after time points was statistically significant.

Since its advancement as a straightforward parameter for visceral adiposity evaluation, LAP has since proved its detection ability for both cardiovascular risk and disease. In a relatively small sample of 210 patients from the general population, LAP was associated with altered lipid profile and elevated diastolic blood pressure [48]. Larger-scale studies with over 50,000 and 95,000 patients showed similar findings, where individuals in the largest-quartile bracket of LAP values had the highest incidence of CVD and all-cause mortality [49,50]. This association remained significant even after controlling potential confounding variables within the general population, with an increase of one unit in LAP translating into a 4-fold hazard risk increase for CVD [50]. Notably, in a cohort of 3000 individuals from the general population without cardiovascular disease diagnosis, LAP demonstrated superior prognostic value for cardiovascular disease incidence over a 10-year follow-up period compared to traditional anthropometric measures and lipid profile laboratory parameters [51]. The clinical usability of LAP extends to the prediction of cardiovascular hospitalization over long-term follow-up in individuals suffering from stable ischemic heart disease [52]. Seriate evaluation of LAP may be of use, as shown by the increased risk for ischemic stroke in patients exposed to a higher LAP over a longer period [53]. The association between LAP, a surrogate marker of abdominal obesity, and all-mortality is partly mediated by inflammation in older adults harboring cardiovascular risks [54].

LAP is a product-derived index, with both parameters expectedly shifting in the same direction after a successful weight loss intervention. Confined to our research protocol, this index has limited use for assessing intervention efficacy, since any reduction in waist circumference and a—proportional or not—improvement in lipid profile would synergically reduce LAP.

For this reason, we developed ACLI, which has not yet been indexed in the specialized literature.

The proposed formula ensures coherence from a physio-pathological point of view. Logarithmic transformation of the ratio ensures robustness of the formula to extreme values, thus decreasing sensitivity to outlier values. The formula also includes a normalization of the index by referring to the protective component of HDL values found in the AIP formula.

We maintained within the formula the correction for waist circumference. Regarding the numerical results, AIP and ACLI have shown a strong inverse correlation. Our hypothesis is that ACLI may have a high prognostic capacity, which needs to be justified by future studies. The cardiovascular prediction potential could be tested in similar interventional studies (focused on weight loss), with long follow-up periods.

### 4.4. Strengths and Limitations

Our study reinforces the beneficial effects of weight loss, translating into a lower risk for cardiovascular or metabolic diseases. This is one of the few research projects to evaluate the impact of weight loss on lipid profile-derived indices, and to our knowledge, this is the first study where the intervention is diet-based. The study setting and initial screening methodology was elaborated around the characteristics of a potential individual fit for a safe and efficient weight loss process. Intervention was strictly controlled for any excessive aerobic exercise in order to ensure uniformity. Statistical analysis was optimized for best accuracy, despite the infrequent detrimental effect of losing the clinical significance of the results (e.g., Spearman correlation). The subgrouping strategy was based on the routine heterogeneity aspects of weight loss.

An important limitation of the study was the broad age range of the included participants (18–65 years). This wide age span may increase data variability and introduce age- and metabolism-related heterogeneity into the study population.

Participants were advised to maintain a moderate level of daily physical activity, defined as 8000–10,000 steps per day, and to avoid intense aerobic exercise. However, physical activity was monitored through self-reported daily step counts, which reflect general activity levels rather than the intensity or type of exercise performed. Therefore, the study did not include strict supervision or objective control of structured aerobic training. This approach allows for the standardization of overall physical activity while minimizing major variations in energy expenditure, but it does not exclude the possibility of variability in exercise intensity among participants.

The planned outcomes for analysis included indexes consistently linked to cardiovascular and metabolic health not yet approved for indiscriminate use, outlining the need for additional research. However, there are limiting aspects to our research. The strict criteria of exclusion may adversely affect the applicability of our results to frail patients. The absence of an additional aerobic exercise program might be detrimental to muscle mass maintenance and endothelial functional status. Regression analysis by ranking in a monotonous non-linear dependency might diminish interpretability. Nevertheless, the restricted choice of non-imaging composite indexes for outcomes out of a vast array of available derived parameters is an inherent limitation by design.

## 5. Conclusions

The findings of this study suggest that the Atherogenic Central Load Index (ACLI) captures key pathophysiological mechanisms linking insulin resistance, chronic inflammation, and atherogenesis. As insulin resistance represents a central driver of endothelial dysfunction, proinflammatory activation, and adverse lipid remodeling, the strong associations observed between ACLI and major inflammatory markers (hs-CRP and IL-6) support its biological relevance within the cardiometabolic continuum. Notably, ACLI demonstrated superior discriminatory capacity for identifying elevated inflammatory status in individuals with overweight and obesity undergoing dietary weight-loss interventions, outperforming established atherogenic indices (AIP, CRI–1, and CRI–2).

These results highlight the potential of ACLI to serve as an integrative marker reflecting the combined burden of dyslipidemia, metabolic dysfunction, and vascular risk. The observed relationships with inflammatory status and cardiovascular risk further support its role as a surrogate indicator of insulin resistance and its downstream proatherogenic effects. Taken together, ACLI may represent a clinically meaningful tool for improved cardiometabolic risk stratification and for monitoring metabolic and vascular responses to lifestyle interventions.

## Figures and Tables

**Table 1:** Anthropometric and biochemical characteristics at the time of enrollment and after 6 months of intervention initiation.

Anthropometric and Biochemical Measurements	Before (at Enrollment)	After (at Study Exit)	*p*-Value *
Age (years), mean ± SD	46.4 ± 9.8	-	
Gender (M/F), n (%)	30/43 (41.1%/58.9%)	-	
Height (m)	1.68 (1.62–1.76)	-	
Weight (Kg)	100.5 (87.7–118.7)	89 (78–102)	0.002
BMI (Kg/m^2^)	34.9 (31.8–41.2)	31.2 (27.9–34.9)	0.001
Waist circumference (cm)	98 (87–107)	89 (80–97)	<0.001
Systolic blood pressure, mmHg	124 (92–189)	112 (84–164)	0.001
Diastolic blood pressure, mmHg	89 (72–121)	71 (54–109)	0.024
Heart rate, bpm	67.2 (72.4–90.2)	63.8 (69–87)	0.018
Biochemical profile			
Fasting blood glucose, (mg/dL)	102 ± 49.8	93.6 ± 41.2	0.001
HbA1c, glycated hemoglobin, %	5.3 (5–5.5)	5.2 (4.9–5.4)	0.003
HDL-C (mg/dL)	47 ± 12.5	45 ± 8.8	0.001
LDL-C (mg/dL)	152 ± 40.8	138 ± 29.6	<0.001
TC (mg/dL)	215 ± 40.9	202 ± 32.2	0.002
Triglycerides (mg/dL)	153 (113–210)	138 (104–178)	0.001
Atherogenic indices			
AIP	0.523 ± 0.227	0.487 ± 0.162	0.046
CRI–1	4.66 (3.77–5.35)	4.60 (3.88–5.26)	0.054
CRI–2	3.28 (2.57–4)	3.22 (2.55–3.71)	0.001
Lipid profile			
LAP	62.6 (38.3–94.6)	40.3 (27.4–64.8)	<0.001
Atherogenic Central Load Index (ACLI)	55.43 (46.85–68.5)	43.27 (35.7–52.5)	0.001
Inflammation markers			
hs-CRP, mg/L	3.76 (2.8–6.2)	2.84 (1.7–3.89)	<0.001
IL-6, pg/mL	2.9 (1.23–4.11)	2.18 (0.8–3.03)	<0.001

Values are n (%), mean ± SD or median (IQR); * t-test for dependent samples or Wilcoxon matched-pairs test were applied. Abbreviations: IQR, interquartile range; SD, standard deviation; BMI, body mass index; HbA1c, glycated hemoglobin; HDL-C, high-density lipoprotein cholesterol; LDL, low-density lipoprotein; TC, total cholesterol; AIP, atherogenic index of plasma; CRI–1, Castelli Risk Indexes–1; CRI–2, Castelli Risk Indexes–2; LAP, lipid accumulation product; ACLI, Atherogenic Central Load Index; hs-CRP, high-sensitivity C-reactive protein; IL-6, interleukin-6.

**Table 2:** Difference in anthropometric and biochemical parameter values at enrollment compared to 6 months after intervention initiation.

Δ, Difference: Post-Intervention and Pre-Intervention	Mean Change, Mean (SD)	95% Confidence Interval of the Mean Change	*p*-Value *
Δ weight, kg	−9.52 (3.45)	−10.33 to −8.72	0.002
Δ weight %, median (IQR)	9.6% (7.3–11.5%)		
Δ BMI	−3.29 (1.16)	−3.56 to −3.02	0.001
Δ waist circumference (cm)	−7.21 (2.69)	−7.63 to −6.37	<0.001
Δ HDL-C (mg/dL)	−4.34 (0.86)	−6.81 to −3.87	0.001
Δ LDL-C (mg/dL)	−19.23 (5.92)	−21.15 to −17.03	<0.001
Δ TC (mg/dL)	−32.16 (7.85)	−38.31 to −27.67	0.002
Δ triglycerides (mg/dL)	−35.41 (16.51)	−40.47 to −32.58	0.001
Δ AIP (atherogenic index of plasma)	−0.09 (0.03)	−0.09 to −0.08	0.046
Δ CRI–1	−0.52 (0.27)	−0.58 to −0.46	0.054
Δ CRI–2	−0.31 (0.15)	−0.35 to −0.28	0.001
Δ LAP	−9.53 (1.92)	−11.17 to −7.89	<0.001
Δ ACLI	−13.92 (6.19)	−15.36 to −12.47	<0.001
Δ hs-CRP, mg/L	−1.16 (0.74)	−1.34 to −0.99	<0.001
Δ IL-6, pg/mL	−0.81 (0.54)	−0.94 to −0.68	<0.001

* t-test for dependent samples or Wilcoxon matched-pairs test were applied. Abbreviations: SD, standard deviation; BMI, body mass index; HDL-C, high-density lipoprotein cholesterol; LDL, low-density lipoprotein; TC, total cholesterol; AIP, atherogenic index of plasma); CRI–1, Castelli Risk Indexes–1; CRI–2, Castelli Risk Indexes–2; LAP, lipid accumulation product; ACLI, Atherogenic Central Load Index; hs-CRP, high-sensitivity C-reactive protein; IL-6, interleukin-6.

**Table 3:** Discriminative accuracy of atherosclerosis-related indices for the inflammatory markers hs-CRP and IL-6 in patients with overweight or obesity undergoing dietary weight-loss interventions.

Test Result Variable (s)	Area Under the Curve (95%CI)	*p*-Value
IL-6 before		
ACLI_pre	0.944 (0.883 to 1.000)	<0.001
AIP_pre	0.871 (0.782 to 0.960)	<0.001
Castelli1_pre	0.721 (0.605 to 0.837)	0.001
Castelli2_pre	0.587 (0.454 to 0.719)	0.203
hs-CRP before		
ACLI_pre	0.947 (0.902 to 0.993)	<0.001
AIP_pre	0.870 (0.784 to 0.957)	<0.001
Castelli1_pre	0.719 (0.602 to 0.836)	0.001
Castelli2_pre	0.635 (0.505 to 0.765)	0.048
IL-6 after		
ACLI_post	0.862 (0.771 to 0.952)	<0.001
AIP_post	0.698 (0.577 to 0.820)	0.005
Castelli1_post	0.691 (0.567 to 0.815)	0.007
Castelli2_post	0.659 (0.528 to 0.791)	0.024
hs-CRP–after		
ACLI_post	0.844 (0.754 to 0.933)	<0.001
AIP_post	0.738 (0.625 to 0.852)	<0.001
Castelli1_post	0.715 (0.597 to 0.833)	0.002
Castelli2_post	0.641 (0.513 to 0.768)	0.039

## Data Availability

The data presented in this study are available from the corresponding author upon reasonable request. The data are not publicly available due to confidentiality reasons.

## Supplementary Materials

The following supporting information can be downloaded at https://www.mdpi.com/article/10.3390/jcm15062321/s1, Table S1: Checklist of items that were included in the prospective cohort study based on the STROBE Statement (https://www.strobe-statement.org/ accessed on 5 February 2026).

## Abbreviations

The following abbreviations are used in this manuscript:

IQR	Interquartile range
SD	Standard deviation
ROC	Receiver operating characteristic
AUC	Area under the curve
BMI	Body mass index
HbA1c	Glycated hemoglobin
HDL-C	High-density lipoprotein cholesterol
LDL	Low-density lipoprotein
TC	Total cholesterol
Δ	Difference
AIP	Atherogenic index of plasma
CRI–1	Castelli Risk Indexes–1
CRI–2	Castelli Risk Indexes–2
LAP	Lipid accumulation product
ACLI	Atherogenic Central Load Index
hs-CRP	High-sensitivity C-reactive protein
IL-6	Interleukin-6
