# Supplementary material for: Impact of a Hypocaloric Diet on Prognostic Biomarkers of Endothelial Dysfunction: A Prospective Study
Source: J Clin Med. 2026 Mar 18;15(6):2321. doi: 10.3390/jcm15062321 (PMC13026628; doi:10.3390/jcm15062321)
Supplement: Supplementary file 1 [file jcm-15-02321-s001.zip › jcm-4149193-supplementary.pdf]

Table S1. Checklist of items that were included in the prospective cohort study based on the STROBE Statement (https://www.strobe-statement.org/)
Prospective observational study: Impact of a hypocaloric diet on prognostic biomarkers of endothelial dysfunction. Prospective study

	Item No.	Recommendation	Page No.
**Title and abstract**	1	(*a*) Indicate the study's design with a commonly used term in the title or the abstract	1
		(*b*) Provide in the abstract an informative and balanced summary of what was done and what was found	1
**Introduction**			
Background/rationale	2	Explain the scientific background and rationale for the investigation being reported	2
Objectives	3	State specific objectives, including any prespecified hypotheses	3
**Methods**			
Study design	4	Present key elements of study design early in the paper	3; Section 2
Setting	5	Describe the setting, locations, and relevant dates, including periods of recruitment, exposure, follow-up, and data collection	4; Section 2
Participants	6	(*a*) *Cohort study*—Give the eligibility criteria, and the sources and methods of selection of participants. Describe methods of follow-up	3-5; Section 2
Variables	7	Clearly define all outcomes, exposures, predictors, potential confounders, and effect modifiers. Give diagnostic criteria, if applicable	5-7; Section 2
Data sources/ measurement	8*	For each variable of interest, give sources of data and details of methods of assessment (measurement). Describe comparability of assessment methods if there is more than one group	5-7; Section 2
Bias	9	Describe any efforts to address potential sources of bias	19
Study size	10	Explain how the study size was arrived at	7 Sample size calculation.

Continued on next page

Quantitative variables	11	Explain how quantitative variables were handled in the analyses. If applicable, describe which groupings were chosen and why	7<br>Statistical analysis
Statistical methods	12	(*a*) Describe all statistical methods, including those used to control for confounding	7<br>Statistical analysis
		(*b*) Describe any methods used to examine subgroups and interactions	
		(*c*) Explain how missing data were addressed	7<br>Statistical analysis
		(*e*) Describe any sensitivity analyses	7<br>Statistical analysis
Results			
Participants	13*	(a) Report numbers of individuals at each stage of study—eg numbers potentially eligible, examined for eligibility, confirmed eligible, included in the study, completing follow-up, and analysed	8<br>Section 3. Results
		(b) Give reasons for non-participation at each stage	8<br>Section 3. Results
		(c) Consider use of a flow diagram	8<br>Section 3. Results
Descriptive data	14*	(a) Give characteristics of study participants (eg demographic, clinical, social) and information on exposures and potential confounders	8-9<br>Section 3. Results
		(b) Indicate number of participants with missing data for each variable of interest	8-9<br>Section 3. Results
		(c) *Cohort study*—Summarise follow-up time (eg, average and total amount)	8-15<br>Section 3. Results
Main results	16		19<br>Section 4. Discussion
		(*b*) Report category boundaries when continuous variables were categorized	

Continued on next page

Other analyses	17	Report other analyses done—eg analyses of subgroups and interactions, and sensitivity analyses	8-15<br>Section 3. Results
**Discussion**			
Key results	18	Summarise key results with reference to study objectives	15-20<br>Section 4.<br>Discussion
Limitations	19	Discuss limitations of the study, taking into account sources of potential bias or imprecision. Discuss both direction and magnitude of any potential bias	19<br>Section 4.<br>Discussion
Interpretation	20	Give a cautious overall interpretation of results considering objectives, limitations, multiplicity of analyses, results from similar studies, and other relevant evidence	15-20<br>Section 4.<br>Discussion
Generalisability	21	Discuss the generalisability (external validity) of the study results	20<br>Section 5.<br>Conclusions
**Other information**			
Funding	22	Give the source of funding and the role of the funders for the present study and, if applicable, for the original study on which the present article is based	-<br>It has no funding.

Discussion

*Give information separately for cases and controls in case-control studies and, if applicable, for exposed and unexposed groups in cohort and cross-sectional studies.

Note: An Explanation and Elaboration article discusses each checklist item and gives methodological background and published examples of transparent reporting. The STROBE checklist is best used in conjunction with this article (freely available on the Web sites of PLoS Medicine at http://www.plosmedicine.org/, Annals of Internal Medicine at http://www.annals.org/, and Epidemiology at http://www.epidem.com/). Information on the STROBE Initiative is available at www.strobe-statement.org.
